# Supplementary material for: ANYI: The ANnotated Yeast Interactome
Source: bioRxiv. 2026 May 5:2026.04.30.721908. Preprint. [Version 1] doi: 10.64898/2026.04.30.721908 (PMC13174366; doi:10.64898/2026.04.30.721908)
Supplement: Supplement 1 [file media-1.pdf]

SUPPLEMENTARY INFORMATION

ANYI: The ANotated Yeast Interactome

Daniel A. Nissley[1,2,*], Muskan Goel[3], Xavier Castellanos-Girouard[4], Charles P. Kuntz[3], Yiqing Wang[5], M. Shahid Mukhtar[5], Adrian Serohijos[4], and Jonathan P. Schlebach[3]

[1]National Synthesis Center for Emergence in the Molecular and Cellular Sciences, National Science Foundation, USA
[2]Institute for Computational and Data Science, Pennsylvania State University, University Park, PA, USA
[3]The James Tarpo Jr. and Margaret Tarpo Department of Chemistry, Purdue University, West Lafayette, IN, USA
[4]Département de Biochimie, Université de Montréal, Montreal, Quebec, Canada
[5]Department of Genetics and Biochemistry, Clemson University, Clemson, SC, USA

*Corresponding Author: dan182@psu.edu

SUPPLEMENTARY METHODS

The 16 sections below correspond to the 16 annotation classes within ANYI. Each methods section corresponds to a set of rows in Table S1 that define the column identifiers in ANYI that can be used to access protein-level information for that annotation class.

To interact with the ANYI database itself, follow the instructions on GitHub to download and install the ANYI Browser Docker container, which includes an example of loading ANYI into Python for analysis.

1 - Network centrality calculations. The Cytoscape (Shannon *et al.* 2003) session file for The Yeast Interactome was downloaded from www.yeast-interactome.org (Michaelis *et al.* 2023) and used as the basis for downstream annotations. This session file was opened in Cytoscape v3.10.3, weighted k-shell decomposition values (Wei *et al.* 2015) calculated with the wk-shell-decomposition app, and the node and edge tables exported. Network models were constructed using these node and edge tables in Python with NetworkX (Hagberg, Schult, and Swart 2008) v3.4.2 and its internal functions used to compute degree centrality, betweenness centrality, eigenvector centrality, closeness centrality, load centrality, and PageRank (with damping coefficient 0.85) for the full network (3,927 nodes). NetworkX was also used to compute information centrality using the largest connected subgraph (3,839 nodes).

2 - Sequence information and sequence-based predictions. Protein sequences (Genome Release 64-5-1) were retrieved from the SGD for the S288C reference genome and associated with network nodes based on their SGD open reading frame identifiers. DeepTMHMM (Hallgren *et al.* 2022) v1.0.24 was used to classify proteins as globular or transmembrane, and SignalP 6.0h fast (Teufel *et al.* 2022) was used to predict signal peptide cleavage sites. Predicted signal peptides were removed based on these cleavage site predictions, and all subsequent analyses used the trimmed sequences.

3 - UniProt annotation. UniProt accession numbers were assigned to each node using the "idmapping" file for S288C yeast from the UniProt FTP site ftp.uniprot.org. Each node was then annotated with information from UniProt (Bateman *et al.* 2025), including post-translational modifications, subcellular localization, Gene Ontology (GO) terms, and protein function information. Gene ontology terms were also converted to a human-readable format using obolibrary (Ashburner *et al.* 2000) and the go-basic.obo reference file (Aleksander et al. 2026). UniProt annotations were extracted from a copy of uniprot_sprot.xml downloaded from the UniProt FTP site ftp.uniprot.org. Copies of all source files are stored on CyVerse (see GitHub).

4 - Intrinsic disorder content and property predictions. Intrinsically disordered regions (IDRs) were predicted from protein sequences using metapredict v3.0 (Lotthammer, Hernández-García *et al.* 2024). Sequence and ensemble properties were computed for all IDRs with CIDER (Holehouse *et al.* 2017) and ALBATROSS (Lotthammer, Ginell *et al.* 2024) functionality within sparrow from github.com/idptools. The fraction of disorder predicted within each protein was

compared to various percentile thresholds computed from the disorder content for all entries for S288C proteins in the DisProt database (Nugnes *et al.* 2026).

5 - Protein stability prediction. Folding free energies were estimated using two approaches for proteins predicted by DeepTMHMM to not be transmembrane proteins: (1) Equation 1 from Ghosh & Dill (2010) (Ghosh and Dill 2010), which depends only on protein length and system temperature (set to 300 K), & (2) the ESM-IF-based model from Cagiada et al. (Cagiada, Ovchinnikov, and Lindorff-Larsen 2025), which requires AlphaFold2-predicted structures (Jumper *et al.* 2021). For this latter calculation, AlphaFold2 structure predictions (version 4) for the yeast proteome were obtained from the European Bioinformatics Institute (EBI) (Varadi *et al.* 2024). For proteins with predicted signal peptides or a mismatch between the sequences of the EBI structure and the SGD reference sequences, new structure predictions were carried out with AlphaFold2. All structures were also scored using Rosetta release 371 with the ref2015 scoring function (Alford et al., 2017). Prior to Rosetta scoring, AlphaFold2 structures were relaxed with the FastRelax protocol in Rosetta using ten independent replicas per protein. Python code to implement the Cagiada et al. 2025 model is based on the example Jupyter Notebook from https://github.com/KULL-Centre/_2024_cagiada_stability.

6 - Protein half-life data integration. Protein half-life information from two large-scale mass spectrometry studies (Christiano *et al.* 2014, Martin-Perez and Villén 2017) was incorporated using simple identifier-based joins. The data from Christiano *et al.* 2014 and Martin-Perez and Villén 2017 were obtained from Table S1 and Data S1, respectively, of the corresponding publications.

7 - Protein expression level data integration. Protein expression levels from a meta study (Ho, Baryshnikova, and Brown 2018) were incorporated using an identifier-based mapping. Specifically, the data from Ho et al. 2018 Table S4, representing the molecules per cell data after correction for autofluorescence, were integrated into ANYI.

8 - Translation efficiency data integration. Pre-computed translation efficiency values for yeast from the sckit-ribo package (Fang *et al.* 2018) were integrated into ANYI. The source file, skr_weinberg_genesTE.csv, is available on the scikit-ribo_manuscript GitHub in the blob/master/Data directory.

9 - Predicting post-translational modifications. In addition to the curated PTM information included from UniProt annotations (see above), yeast phosphorylation sites from PTMeXchange mass spectrometry data (PXD071918) downloaded from ProteomeXchange (Deutsch *et al.* 2023) were associated with each network node. In addition, the locations of 19 classes of PTMs were predicted with the PTMGPT2 language model (Shrestha *et al.* 2024).

10 - Entanglement status data integration. Binary entanglement status information computed from both AlphaFold2 and experimental structures was included for each protein based on results from Rana & Sitarik *et al.* 2024. Here, entanglements are defined as non-covalent lasso structures (Rana *et al.* 2024).

11 - Chaperone interaction data integration. The list of chaperones and co-chaperones in yeast from Rizzolo *et al.* 2017 Table S1 was used to define a list of chaperone nodes (Rizzolo *et al.* 2017). All nodes that share an edge with a chaperone node in The Yeast Interactome are considered to interact with that chaperone.

12 - Oligomerization state data integration. The Complex Portal (Balu *et al.* 2025) reference file for yeast, 59292.tsv, was downloaded from EBI and parsed to extract information at the individual protein level. Within ANYI, various binary columns encode if each protein participates in the formation of specific types of complexes (*e.g.*, homodimer, homotrimer, heterodimer) or in the formation of complexes with unknown stoichiometry.

13 - Domain annotations. The InterPro (Blum *et al.* 2025) database file protein2ipr.dat.gz was downloaded from EBI and parsed to extract domain annotations for each protein in ANYI (see Table S1 for a summary of all domain annotation methods included). In addition to a list of the domains identified by each method, we also include a summary of the number of domains identified for each protein by each method.

14 - Essentiality data integration. The SGD web browser was used to select all S288C proteins with a phenotype of "inviable", which indicates proteins that are essential to growth, and these annotations then mapped to network nodes.

15 - Yeast two-hybrid PPI data integration. Data were obtained from the CCSB Interactome Database (Yu *et al.* 2008) and network centrality calculations carried out as described for The Yeast Interactome data in section **1 - Network centrality calculations**. The network centralities within the yeast two-hybrid interactome were associated with each node within the mass spectrometry-derived Yeast Interactome from the Mann Lab to provide a secondary set of reference centralities.

16 - Thermal proteome profiling data integration. Proteome-wide thermal stability information was incorporated from the Meltome Atlas (Jarzab *et al.* 2020). Coefficient of determination values for melting curve fit line evaluation are reproduced in ANYI to enable the filtering of results by quality.

Supplementary Table 1 – Annotations within ANYI

Annotation Class	Index	Data Column Name	Explanation
0 - Identifiers	1	node	SGD systematic name; primary identifier
	2	UniProtKB-AC	UniProt knowledge base primary accession; secondary identifier
1 - Network centrality	3	_wkshell	Weighted *k*-shell decomposition value for this node
	4	degree_centrality	Degree centrality of this node
	5	betweenness_centrality	Betweenness centrality of this node
	6	eigenvector_centrality	Eigenvector centrality of this node
	7	closeness_centrality	Closeness centrality of this node
	8	load_centrality	Load centrality of this node
	9	pagerank	PageRank of this node
	10	information_centrality	Information centrality of this node
2 - Sequence information and sequence-based predictions	11	has_verified_sequence	Boolean True if this node could be associated with an SGD Open Reading Frame, otherwise False
	12	sequence	The single-letter amino acid sequence of this protein from SGD
	13	DeepTMHMM_trimmed_sequence	Trimmed single-letter amino acid sequence resulting from removal of residues identified by DeepTMHMM to be part of signal sequence (S in output mask)
	14	DeepTMHMM_class	One from the list of GLOB (globular), TM (transmembrane, alpha helical), SP (has signal sequence), BETA (transmembrane, beta barrel), or SP+TM (alpha helical membrane protein with signal sequence)
	15	cleavage_site_start	Predicted start site of signal sequence cleavage from SignalP; if this value is i, cleavage is between i and i + 1
	16	signalP_trimmed_sequence	Sequence predicted by SignalP to remain after cleavage of the signal peptide; if there was no signal sequence, this is identical to sequence
3 - UniProt annotations	17	ProteinName	Primary name of the protein for this node from UniProt
	18	GO_terms	GO terms for this protein from UniProt
	19	GO_term_human_readable	GO terms for this protein from UniProt with their human-readable name included
	20	localization_keywords	Localization information from UniProt (UniProt annotation)
	21	parsed_functions	Functional information from UniProt
	22	parsed_PTMs	Post-translation modifications from UniProt annotations

Supplementary Table 1 (cont.) – Annotations within ANYI

Annotation Class	Index	Data Column Name	Explanation
4 - Disorder predictions	23	IDR_count	Integer number of discrete IDRs within the protein using a 30-aa cutoff
	24	IDR_sequences	Dictionary containing all IDR sequences (not just those meeting the 30 aa cutoff); keys are integers, matches IDR_ranges. Computed with metapredict v3 on the signalP_trimmed_sequence
	25	IDR_ranges	Dictionary containing IDR residues ranges for all IDR sequences (keys are integers, matches IDR_sequences)
	26	N_aa_disordered	Integer number of disordered residues in the protein
	27	disorder_fraction	Fraction of disordered residues in the protein
	28	is_disordered_0.05	1 if disorder_fraction is ≥ the 5th percentile of disorder fraction within DisProt for S288C proteins, otherwise 0
	29	is_disordered_0.10	1 if disorder_fraction is ≥ the 10th percentile of disorder fraction within DisProt for S288C proteins, otherwise 0
	30	is_disordered_0.15	1 if disorder_fraction is ≥ the 15th percentile of disorder fraction within DisProt for S288C proteins, otherwise 0
	31	is_disordered_0.20	1 if disorder_fraction is ≥ the 20th percentile of disorder fraction within DisProt for S288C proteins, otherwise 0
	32	is_disordered_0.25	1 if disorder_fraction is ≥ the 25th percentile of disorder fraction within DisProt for S288C proteins, otherwise 0
	33	is_disordered_0.30	1 if disorder_fraction is ≥ the 30th percentile of disorder fraction within DisProt for S288C proteins, otherwise 0
	34	is_disordered_0.35	1 if disorder_fraction is ≥ the 35th percentile of disorder fraction within DisProt for S288C proteins, otherwise 0
	35	is_disordered_040	1 if disorder_fraction is ≥ the 40th percentile of disorder fraction within DisProt for S288C proteins, otherwise 0
	36	is_disordered_0.45	1 if disorder_fraction is ≥ the 45th percentile of disorder fraction within DisProt for S288C proteins, otherwise 0
	37	is_disordered_0.50	1 if disorder_fraction is ≥ the 50th percentile of disorder fraction within DisProt for S288C proteins, otherwise 0
	38	is_disordered_0.55	1 if disorder_fraction is ≥ the 55th percentile of disorder fraction within DisProt for S288C proteins, otherwise 0
	39	is_disordered_0.60	1 if disorder_fraction is ≥ the 60th percentile of disorder fraction within DisProt for S288C proteins, otherwise 0
	40	is_disordered_0.65	1 if disorder_fraction is ≥ the 65th percentile of disorder fraction within DisProt for S288C proteins, otherwise 0
	41	is_disordered_0.70	1 if disorder_fraction is ≥ the 70th percentile of disorder fraction within DisProt for S288C proteins, otherwise 0
	42	is_disordered_0.75	1 if disorder_fraction is ≥ the 75th percentile of disorder fraction within DisProt for S288C proteins, otherwise 0
	43	is_disordered_0.80	1 if disorder_fraction is ≥ the 80th percentile of disorder fraction within DisProt for S288C proteins, otherwise 0
	44	is_disordered_0.85	1 if disorder_fraction is ≥ the 85th percentile of disorder fraction within DisProt for S288C proteins, otherwise 0
	45	is_disordered_0.90	1 if disorder_fraction is ≥ the 90th percentile of disorder fraction within DisProt for S288C proteins, otherwise 0
	46	is_disordered_0.95	1 if disorder_fraction is ≥ the 95th percentile of disorder fraction within DisProt for S288C proteins, otherwise 0
	47	is_disordered_1.00	1 if disorder_fraction is ≥ the 100th percentile of disorder fraction within DisProt for S288C proteins, otherwise 0
	48	albatross	Dictionary containing predicted IDR conformational ensemble properties (keys are integers, matches IDR_sequences)
	49	cider	Dictonary containing IDR sequence properties (keys are integers, matches IDR_sequences)

Supplementary Table 1 (cont.) – Annotations within ANYI

Annotation Class	Index	Data Column Name	Explanation
5 - Predicted protein structure & stability	50	structure_sequence	Amino acid sequence from EBI AlphaFold2 structure as a string
	51	cleaved_structure_sequence	Sequence for this protein after removal of signal sequence
	52	sequence_matches_structure	TRUE if the sequence from the EBI AlphaFold2 structure matches the sequence from SGD, otherwise FALSE
	53	final_sequence	Final amino acid sequence matching the final structure
	54	final_structure_source	Source of the final structure selected from the list: {EBI, AF2-cleaved, AF2, None}. EBI = the original structure from the EBI AlphaFold2 database; AF2-cleaved = the original structure from the EBI with the signal sequence removed; AF2 = a new AlphaFold2 prediction; None = No structure available (typically due to a lack of a verified sequence)
	55	mean_plddt	Average of C-alpha pLDDT values for all proteins with a structure
	56	L	Length of the final_sequence for each protein
	57	Ghosh-Dill-dG	Free energy difference between folded and unfolded states for this protein calculated with Eq. 1 of Ghosh-Dill 2010 (kcal/mol)
	58	cagiada-dG	Absolute free energy difference between folded and unfolded states for this protein calculated with Cagiada language model (kcal/mol)
	59	Rosetta_total_score_0001	Value of Rosetta total_score with the ref2015 scoring function for the first FastRelax replicate
	60	Rosetta_total_score_0002	Value of Rosetta total_score with the ref2015 scoring function for the second FastRelax replicate
	61	Rosetta_total_score_0003	Value of Rosetta total_score with the ref2015 scoring function for the third FastRelax replicate
	62	Rosetta_total_score_0004	Value of Rosetta total_score with the ref2015 scoring function for the fourth FastRelax replicate
	63	Rosetta_total_score_0005	Value of Rosetta total_score with the ref2015 scoring function for the fifth FastRelax replicate
	64	Rosetta_total_score_0006	Value of Rosetta total_score with the ref2015 scoring function for the sixth FastRelax replicate
	65	Rosetta_total_score_0007	Value of Rosetta total_score with the ref2015 scoring function for the seventh FastRelax replicate
	66	Rosetta_total_score_0008	Value of Rosetta total_score with the ref2015 scoring function for the eighth FastRelax replicate
	67	Rosetta_total_score_0009	Value of Rosetta total_score with the ref2015 scoring function for the ninth FastRelax replicate
	68	Rosetta_total_score_0010	Value of Rosetta total_score with the ref2015 scoring function for the tenth FastRelax replicate
	69	Rosetta_best_total_score	Best (lowest) value of Rosetta total_score with the ref2015 scoring function across all 10 FastRelax replicates
	70	Rosetta_best_pose_index	Index of pose with best total_score

Supplementary Table 1 (cont.) – Annotations within ANYI

Annotation Class	Index	Data Column Name	Description
6 - Protein half life	71	Christiano_degradation_rate(min-1)	Degradation rate in inverse minutes from Christiano et al. 2014
	72	Christiano_degradation_R2	Pearson correlation coefficient from Christiano et al. 2014 curve fitting
	73	Christiano_halflife_min	Halflife in minutes from Christiano et al. 2014
	74	Villen_halflife_hours	Halflife in hours from Martin-Perez and Villen 2017
	75	Villen_halflife_SD_hours	Halflife standard deviation in hours from Martin-Perex and Villen 2017
	76	Villen_halflife_CV	Halflife coefficient of variation from Martin-Perex and Villen 2017
	77	Villen_halflife_min	Halflife in minutes from Martin-Perex and Villen 2017
	78	Villen_halflife_SD_min	Halflife standard deviation in minutes from Martin-Perex and Villen 2017
7 - Protein expression level	79	mean_molecules_per_cell	Mean molecules per cell from Ho et al. 2018
	80	median_molecules_per_cell	Median molecules per cell from Ho et al. 2018
	81	expression_CV	Coefficient of variation of expression measurements from Ho et al. 2018
8 - Protein translation efficiency	82	log2_TE	log base 2 of translation efficiency from SciKit-ribo for Weinberg 2016 Ribo-Seq data
9 - Post-translational modifications	83	ptm_bronze	Phosphorylation sites with "Bronze" evidence from PTMeXchange
	84	ptm_gold	Phosphorylation sites with "Gold" evidence from PTMeXchange
	85	ptm_silver	Phosphorylation sites with "Silver" evidence from PTMeXchange
	86	Acetylation (K)	List of lysine residue numbers that are predicted by PTMGPT2 to be acetylated
	87	Amidation (V)	List of valine residue numbers that are predicted by PTMGPT2 to be amidated
	88	Formylation (K)	List of lysine residue numbers that are predicted by PTMGPT2 to be formylated
	89	Glutarylation (K)	List of lysine residue numbers that are predicted by PTMGPT2 to be glutarylated
	90	Glutathionylation (C)	List of cysteine residue numbers that are predicted by PTMGPT2 to be glutathionylated
	91	Hydroxylation (K)	List of lysine residue numbers that are predicted by PTMGPT2 to be hydroxylated
	92	Hydroxylation (P)	List of proline residue numbers that are predicted by PTMGPT2 to be hydroxylated
	93	Malonylation (K)	List of lysine residue numbers that are predicted by PTMGPT2 to be malonylated
	94	Methylation (K)	List of lysine residue numbers that are predicted by PTMGPT2 to be methylated
	95	Methylation (R)	List of arginine residue numbers that are predicted by PTMGPT2 to be methylated
	96	N-linked Glycosylation (N)	List of asparagine residue numbers that are predicted by PTMGPT2 to be N-glycosylated
	97	O-linked Glycosylation (S,T)	List of serine or threonine residue numbers that are predicted by PTMGPT2 to be O-glycosylated
	98	Phosphorylation (S,T)	List of serine or threonine residue numbers that are predicted by PTMGPT2 to be phosphorylated
	99	Phosphorylation (Y)	List of tyrosine residue numbers that are predicted by PTMGPT2 to be phosphorylated
	100	S-nitrosylation (C)	List of cysteine residue numbers that are predicted by PTMGPT2 to be S-nitrosylated
	101	S-palmitoylation (C)	List of cysteine residue numbers that are predicted by PTMGPT2 to be S-palmitoylated

Supplementary Table 1 (cont.) – Annotations within ANYI

Annotation Class	Index	Data Column Name	Description
9 - Post-translational modifications	102	Succinylation (K)	List of lysine residue numbers that are predicted by PTMGPT2 to be succinylated
	103	Sumoylation (K)	List of lysine residue numbers that are predicted by PTMGPT2 to be sumoylated
	104	Ubiquitination (K)	List of lysine residue numbers that are predicted by PTMGPT2 to be ubiquitinated
10 - Entanglement status	105	AF2_contains_entanglement	1 if AlphaFold2 structure for this protein contains an entanglement, otherwise 0. If a protein did not have its entanglement assessed, its value will be 0
	106	exp_contains_entanglement	1 if experimental structure for this protein contains an entanglement, otherwise 0. If a protein did not have its entanglement assessed, its value will be 0
11 - Chaperone interactions	107	interacting_chaperones	List of chaperones/co-chaperones with which this protein interacts
	108	interacting_chaperone_info	Additional information on the chaperone family, etc.
12 - Oligomerization state	109	homodimer	1 if protein is reported in Complex Portal to form a homodimer, otherwise 0
	110	homotrimer	1 if protein is reported in Complex Portal to form a homotrimer, otherwise 0
	111	homotetramer	1 if protein is reported in Complex Portal to form a homotetramer, otherwise 0
	112	heterodimer	1 if protein is reported in Complex Portal to form a heterodimer, otherwise 0
	113	heterotrimer	1 if protein is reported in Complex Portal to form a heterotrimer, otherwise 0
	114	heterotetramer	1 if protein is reported in Complex Portal to form a heterotetramer, otherwise 0
	115	heteropentamer	1 if protein is reported in Complex Portal to form a heteropentamer, otherwise 0
	116	heterohexamer	1 if protein is reported in Complex Portal to form a heterohexamer, otherwise 0
	117	heteroheptamer	1 if protein is reported in Complex Portal to form a heteroheptamer, otherwise 0
	118	heterooctamer	1 if protein is reported in Complex Portal to form a heterooctamer, otherwise 0
	119	heterononamer	1 if protein is reported in Complex Portal to form a heterononamer, otherwise 0
	120	heterodecamer	1 if protein is reported in Complex Portal to form a heterodecamer, otherwise 0
	121	in_complex	1 if protein is reported in Complex Portal to participate in formation of at least 1 complex, otherwise 0
	122	complex_partners	List of other proteins that form stable complexes with this node
	123	complex_count_unknown	Integer number of complexes with unknown stoichiometry in which this protein is found
	124	complex_count	Integer number of complexes of known or unknown stoichiometry in which this protein is found
13 - Domain annotations	125	PROSITE_PROFILE_Ndomains	Number of PROSITE PROFILE domains from InterPro for this protein
	126	PROSITE_PROFILE_domains	PROSITE PROFILE domain ranges for this protein
	127	Gene3D_Ndomains	Number of Gene3DD domains from InterPro for this protein
	128	Gene3D_domains	Gene3D domain ranges for this protein
	129	PANTHER_Ndomains	Number of PANTHER domains from InterPro for this protein
	130	PANTHER_domains	PANTHER domain ranges for this protein
	131	Pfam_Ndomains	Number of Pfam domains from InterPro for this protein

Supplementary Table 1 (cont.) – Annotations within ANYI

Annotation Class	Index	Data Column Name	Description
	132	Pfam_domains	Pfam domain ranges for this protein
	133	SUPERFAMILY_Ndomains	Number of superfamily domains from InterPro for this protein
	134	SUPERFAMILY_domains	Superfamily domain ranges for this protein
	135	PROSITE_PATTERN_Ndomains	Number of PROSITE PATTERN domains from InterPro for this protein
	136	PROSITE_PATTERN_domains	PROSITE PATTERN domain ranges for this protein
	137	SMART_Ndomains	Number of SMART domains from InterPro for this protein
	138	SMART_domains	SMART domain ranges for this protein
	139	TIGRFAM_Ndomains	Number of TIGRFAM domains from InterPro for this protein
	140	TIGRFAM_domains	TIGRFAM domain ranges for this protein
14 - Essentiality	141	essential	1 if this protein is essential, 0 if it is not (from SGD function browser)
	142	essentiality_details	Detail of the essentiality finding
15 - Yeast two-hybrid network data	143	Y2H_wkshell	Weighted k-shell decomposition result; computed with Cytoscape app wk-shell-decomposition
	144	Y2H_degree_centrality	Degree centrality; computed with NetworkX
	145	Y2H_betweenness_centrality	Betweenness centrality; computed with NetworkX
	146	Y2H_eigenvector_centrality	Eigenvector centrality; computed with NetworkX
	147	Y2H_closeness_centrality	Closeness centrality; computed with NetworkX
	148	Y2H_load_centrality	Load centrality; computed with NetworkX
	149	Y2H_pagerank	PageRank; computed with NetworkX with damping parameter = 0.85
	150	Y2H_information_centrality	Information centrality; computed with NetworkX on the largest connected subgraph
16 - Thermal proteome profiling	151	meltome-melting-point	Meltome Atlas melting temperature in degrees Celsius
	152	meltome-stability-class	Meltome Atlas stability class for this protein from the list {non-melter, stable, medium, non-stable}
	153	meltome-AUC	Area under the melting curve for this protein in Meltome Atlas
	154	meltome-fit-converged	True if the Meltome Atlas fit converged for this protein, otherwise np.nan
	155	meltome-fit-R2	Pearson correlation coefficient for melting curve fit for this protein

REFERENCES

Ashburner M, Ball CA, Blake JA *et al.* Gene Ontology: tool for the unification of biology. *Nat Genet* 2000;**25**:25–9. https://doi.org/10.1038/75556.

Balu S, Huget S, Medina Reyes JJ *et al.* Complex portal 2025: Predicted human complexes and enhanced visualisation tools for the comparison of orthologous and paralogous complexes. *Nucleic Acids Res* 2025;**53**(D1):D644–50. https://doi.org/10.1093/nar/gkae1085.

Bateman A, Martin MJ, Orchard S *et al.* UniProt: the Universal Protein Knowledgebase in 2025. *Nucleic Acids Res* 2025;**53**(D1):D609–17. https://doi.org/10.1093/nar/gkae1010.

Blum M, Andreeva A, Florentino LC *et al.* InterPro: The protein sequence classification resource in 2025. *Nucleic Acids Res* 2025;**53**(D1):D444–56. https://doi.org/10.1093/nar/gkae1082.

Cagiada M, Ovchinnikov S, Lindorff-Larsen K. Predicting absolute protein folding stability using generative models. *Protein Science* 2025;**34**(1). https://doi.org/10.1002/pro.5233.

Christiano R, Nagaraj N, Fröhlich F *et al.* Global Proteome Turnover Analyses of the Yeasts S. cerevisiae and S. pombe. *Cell Rep* 2014;**9**(5):1959–65. https://doi.org/10.1016/j.celrep.2014.10.065.

Deutsch EW, Bandeira N, Perez-Riverol Y *et al.* The ProteomeXchange consortium at 10 years: 2023 update. *Nucleic Acids Res* 2023;**51**(D1):D1539–48. https://doi.org/10.1093/nar/gkac1040.

Fang H, Huang YF, Radhakrishnan A *et al.* Scikit-ribo Enables Accurate Estimation and Robust Modeling of Translation Dynamics at Codon Resolution. *Cell Syst* 2018;**6**(2):180-191.e4. https://doi.org/10.1016/j.cels.2017.12.007.

Ghosh K, Dill K. Cellular Proteomes Have Broad Distributions of Protein Stability. *Biophys J* 2010;**99**(12):3996–4002. https://doi.org/10.1016/j.bpj.2010.10.036.

Hagberg A, Schult D, Swart P. Exploring Network Structure, Dynamics, and Function using NetworkX. *Proceedings of the 7th Python in Science Conference (SciPy2008)* Aug. 2008:11–6. https://doi.org/10.25080/TCWV9851.

Hallgren J, Tsirigos KD, Pedersen MD *et al.* DeepTMHMM predicts alpha and beta transmembrane proteins using deep neural networks. *BioRxiv* 10 Apr. 2022:1–10. https://doi.org/10.1101/2022.04.08.487609.

Ho B, Baryshnikova A, Brown GW. Unification of Protein Abundance Datasets Yields a Quantitative Saccharomyces cerevisiae Proteome. *Cell Syst* 2018;**6**(2):192-205.e3. https://doi.org/10.1016/j.cels.2017.12.004.

Holehouse AS, Das RK, Ahad JN *et al.* CIDER: Resources to Analyze Sequence-Ensemble Relationships of Intrinsically Disordered Proteins. *Biophys J* 2017;**112**(1):16–21. https://doi.org/10.1016/j.bpj.2016.11.3200.

Jarzab A, Kurzawa N, Hopf T *et al.* Meltome atlas—thermal proteome stability across the tree of life. *Nat Methods* 2020;**17**(5):495–503. https://doi.org/10.1038/s41592-020-0801-4.

Jumper J, Evans R, Pritzel A *et al.* Highly accurate protein structure prediction with AlphaFold. *Nature* 2021;**596**(7873):583–9. https://doi.org/10.1038/s41586-021-03819-2.

Lotthammer JM, Ginell GM, Griffith D *et al.* Direct prediction of intrinsically disordered protein conformational properties from sequence. *Nat Methods* 2024;**21**(3):465–76. https://doi.org/10.1038/s41592-023-02159-5.

Lotthammer JM, Hernández-García J, Griffith D *et al.* Metapredict enables accurate disorder prediction across the Tree of Life. *BioRxiv* published online 7 Nov. 2024. https://doi.org/10.1101/2024.11.05.622168.

Martin-Perez M, Villén J. Determinants and Regulation of Protein Turnover in Yeast. *Cell Syst* 2017;**5**(3):283-294.e5. https://doi.org/10.1016/j.cels.2017.08.008.

Michaelis AC, Brunner AD, Zwiebel M *et al.* The social and structural architecture of the yeast protein interactome. *Nature* 2023;**624**(7990):192–200. https://doi.org/10.1038/s41586-023-06739-5.

Nugnes MV, Bouhraoua KEA, Zoubiri M *et al.* DisProt in 2026: enhancing intrinsically disordered proteins accessibility, deposition, and annotation. *Nucleic Acids Res* 2026;**54**(D1):D383–92. https://doi.org/10.1093/nar/gkaf1175.

Rana V, Sitarik I, Petucci J *et al.* Non-covalent Lasso Entanglements in Folded Proteins: Prevalence, Functional Implications, and Evolutionary Significance. *J Mol Biol* 2024;**436**(6). https://doi.org/10.1016/j.jmb.2024.168459.

Rizzolo K, Huen J, Kumar A *et al.* Features of the Chaperone Cellular Network Revealed through Systematic Interaction Mapping. *Cell Rep* 2017;**20**(11):2735–48. https://doi.org/10.1016/j.celrep.2017.08.074.

Shannon P, Markiel A, Ozier O *et al.* Cytoscape: A software environment for integrated models of biomolecular interaction networks. *Genome Res* 2003;**13**(11):2498–504. https://doi.org/10.1101/gr.1239303.

Shrestha P, Kandel J, Tayara H *et al.* Post-translational modification prediction via prompt-based fine-tuning of a GPT-2 model. *Nat Commun* 2024;**15**(1). https://doi.org/10.1038/s41467-024-51071-9.

Teufel F, Almagro Armenteros JJ, Johansen AR *et al.* SignalP 6.0 predicts all five types of signal peptides using protein language models. *Nat Biotechnol* 2022;**40**(7):1023–5. https://doi.org/10.1038/s41587-021-01156-3.

Varadi M, Bertoni D, Magana P *et al.* AlphaFold Protein Structure Database in 2024: providing structure coverage for over 214 million protein sequences. *Nucleic Acids Res* 2024;**52**(D1):D368–75. https://doi.org/10.1093/nar/gkad1011.

Wei B, Liu J, Wei D *et al.* Weighted k-shell decomposition for complex networks based on potential edge weights. *Physica A* 2015;**420**:277–83. https://doi.org/10.1016/j.physa.2014.11.012.

Yu H, Braun P, Yildirim M *et al.* High-Quality Binary Protein Interaction Map of the Yeast Interactome Network. *Science (1979)* 2008;**322**(5898):104–10. https://doi.org/10.1126/science.1161847.
